# Baseline serum albumin level as a predictive factor for the efficacy of trifluridine/tipiracil plus bevacizumab in metastatic colorectal cancer: a retrospective cohort study

**DOI:** 10.1186/s40780-025-00518-2

**Published:** 2025-12-03

**Authors:** Masatoshi Maki, Ryo Takada, Haruka Sumii, Miki Fujiwara, Hisashi Tagashira, Okura Yusuke

**Affiliations:** 1 Department of Hospital Pharmacy, National Hospital Organization Okayama Medical Center, Okayama, Japan; 2 Department of Hospital Pharmacy, National Hospital Organization Fukuyama Medical Center, Fukuyama, Japan; 3 Department of Hospital Pharmacy, Okayama Medical Center, 1711-1 Tamasu, Kita-ku, Okayama-City, Okayama, 701-1192 Japan

**Keywords:** Albumin, Colorectal cancer, Trifluridine/tipiracil, Bevacizumab, Predictive marker

## Abstract

**Background:**

Trifluridine/tipiracil (FTD/TPI) combined with bevacizumab (BEV) has become a standard later-line therapy for metastatic colorectal cancer (mCRC). However, predictive biomarkers of treatment efficacy remain limited. Serum albumin (Alb)—reflecting nutritional and inflammatory status—has been reported as a prognostic factor in various malignancies, but its predictive value in patients receiving FTD/TPI plus BEV is unclear. We examined whether baseline Alb levels are linked to treatment outcomes in patients with metastatic CRC receiving FTD/TPI plus BEV, aiming to clarify if Alb could serve as a predictive marker of therapeutic efficacy.

**Methods:**

We retrospectively analyzed patients with unresectable or recurrent mCRC treated with FTD/TPI plus BEV at Fukuyama Medical Center between December 2017 and March 2024. Patients were divided into High- or Low-Alb groups based on an optimal cutoff derived from receiver operating characteristic (ROC) analysis for progression-free survival (PFS). The primary endpoint was PFS, and the secondary endpoint was overall survival (OS). Survival outcomes were assessed using the Kaplan–Meier method and Cox proportional hazards models.

**Results:**

Sixty-nine patients were included (median age, 69 years). ROC analysis identified an Alb cutoff of 3.7 g/dL (area under the curve: 0.740). Using this cutoff, 39 patients (56.5%) were included in the High-Alb group. Patients in the High-Alb group had significantly lower lactate dehydrogenase (LDH) and C-reactive protein levels than those in the Low-Alb group. The median PFS (5.2 vs. 3.0 months; *p* < 0.01) and OS (15.6 vs. 6.0 months; *p* < 0.01) were significantly longer in the High-Alb group than in the Low-Alb group. In the multivariate analysis, Alb ≥3.7 g/dL was independently associated with improved PFS (hazard ratio [HR]: 0.40, 95% confidence interval [CI]: 0.22–0.73, *p* = 0.003), whereas LDH ≥338 U/L was associated with shorter PFS (HR: 2.31, 95% CI: 1.28–4.32, *p* = 0.009).

**Conclusions:**

Baseline serum Alb levels were associated with survival outcomes in patients with mCRC treated with FTD/TPI plus BEV. Thus, Alb may represent a simple and clinically accessible marker with potential predictive value. Initiating FTD/TPI plus BEV before a substantial decline in nutritional or inflammatory status may help achieve more favorable outcomes.

## Background

Colorectal cancer (CRC) is one of the most prevalent malignancies worldwide and is associated with high incidence and mortality rates [1, 2]. For patients with advanced, recurrent, or unresectable disease, systemic chemotherapy remains the cornerstone of treatment. In recent years, trifluridine/tipiracil (FTD/TPI) has demonstrated a survival benefit in the later-line setting [3]. Moreover, the addition of bevacizumab (BEV) to FTD/TPI has been demonstrated to further improve both progression-free survival (PFS) and overall survival (OS) [4, 5]. Based on these findings, FTD/TPI plus BEV has become widely adopted as a standard later-line therapy for advanced or recurrent CRC [6, 7].

Despite this progress, the clinical efficacy of FTD/TPI plus BEV varies considerably among patients, and reliable biomarkers to predict treatment outcomes have not yet been fully established. Routine blood tests provide valuable insights into both the general condition of patients and tumor biology [8–10]. Among these, serum albumin (Alb) is a simple and practical marker that reflects nutritional and inflammatory status as well as overall prognosis, and it has been reported as a prognostic factor in various malignancies [11, 12]. Low Alb levels are often accompanied by elevated C-reactive protein (CRP) or lactate dehydrogenase (LDH) levels, suggesting systemic inflammation or increased tumor activity. CRP has been associated with cancer-related inflammation and poor outcomes [13], whereas LDH reflects tumor metabolism and burden [14, 15], with an elevated LDH level recognized as an unfavorable prognostic factor for CRC [16, 17].

In addition, inflammatory indices such as the neutrophil-to-lymphocyte ratio (NLR) and lymphocyte-to-monocyte ratio have been proposed as potential prognostic markers in patients treated with FTD/TPI or FTD/TPI plus BEV [18, 19]. Similarly, Alb—as an inflammation-based indicator—has been linked to shorter OS and PFS in CRC [20]. However, its predictive value in the specific context of FTD/TPI plus BEV therapy remains unclear.

Therefore, we aimed to retrospectively investigate the association between baseline Alb levels and treatment outcomes in patients with metastatic CRC receiving FTD/TPI plus BEV, with the aim of determining whether Alb may serve as a predictive factor for its therapeutic efficacy.

## Methods

### Study design and patient population

**Fig. 1:**
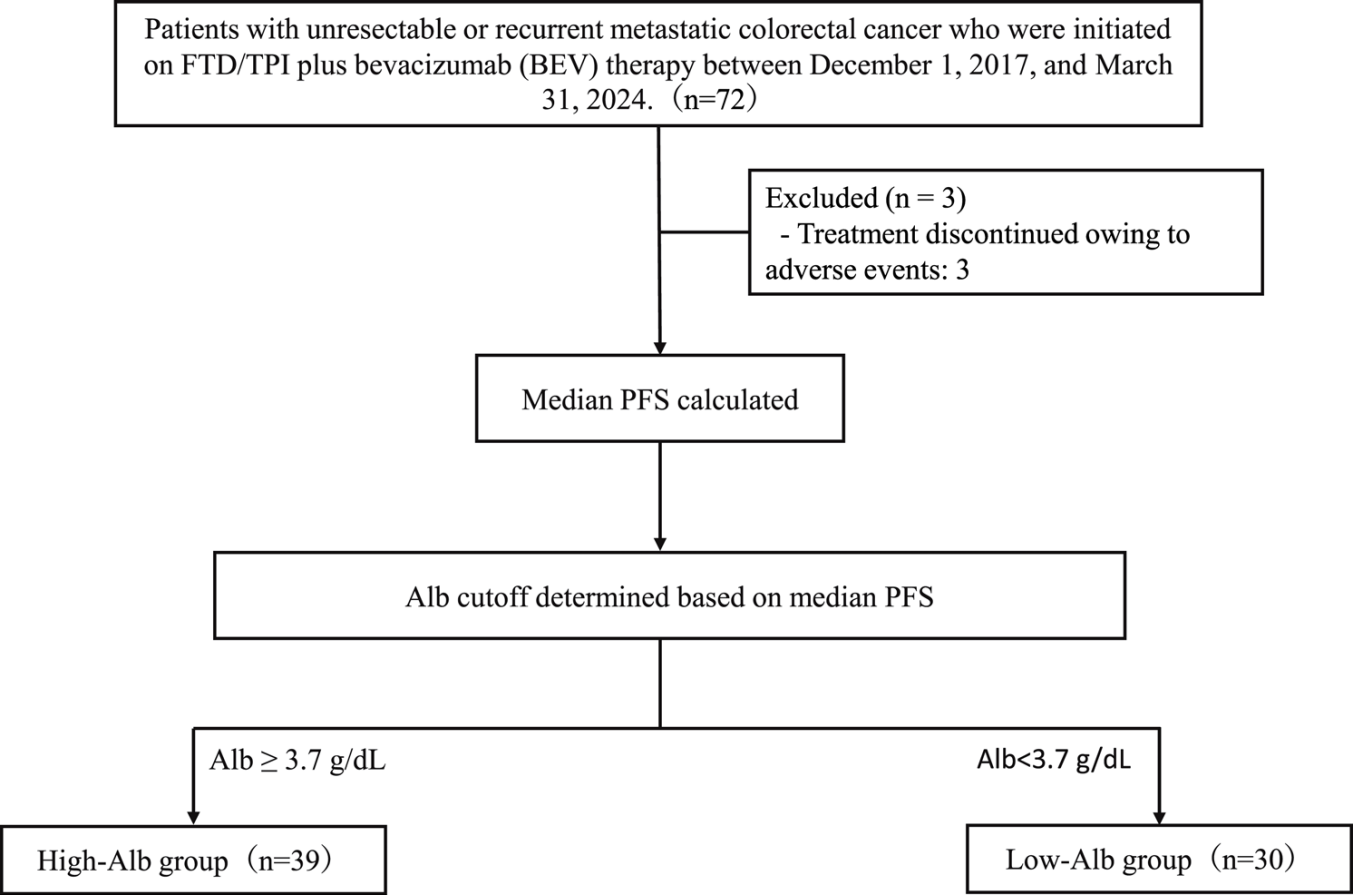
Flowchart of patient selection

In this single-center, retrospective cohort study, we evaluated the association between baseline serum Alb levels and the efficacy of FTD/TPI plus BEV in patients with metastatic mCRC. Data were collected at Fukuyama Medical Center, Hiroshima, Japan, from December 1, 2017, to March 31, 2024. A total of 72 patients who received FTD/TPI plus BEV during this period were analyzed. The study protocol is illustrated in Fig. 1.

Patients with unresectable or recurrent mCRC who had received first-, second-, or third-line chemotherapy and subsequently experienced disease progression were treated with FTD/TPI plus BEV. Patients who discontinued treatment owing to adverse events were excluded from the analysis. FTD/TPI was administered orally at a dose of 35 mg/m^2^ twice daily according to one of the following two schedules: the standard schedule (days 1–5 and 8–12 of a 28-day cycle) or the biweekly schedule (days 1–5 and 15–19 of a 28-day cycle). Bevacizumab was administered intravenously at 5 mg/kg on days 1 and 15 of each cycle.

No formal sample size calculation was performed because this was an exploratory retrospective study that included all eligible patients treated with FTD/TPI plus BEV at our institution during the study period.

Patients were categorized into High-Alb and Low-Alb groups based on baseline serum Alb levels. The cutoff value for Alb was determined by receiver operating characteristic (ROC) curve analysis using PFS as the reference, with the median PFS of the study population as the anchor point. Patients with Alb levels equal to or above this cutoff were assigned to the High-Alb group and those below the cutoff were assigned to the Low-Alb group.

### Data collection

The following data were extracted from the medical records of patients: age, sex, Eastern Cooperative Oncology Group performance status (ECOG-PS), body mass index, mutation status, metastatic sites, primary tumor location, history of primary tumor resection, prior treatments, treatment line, initial FTD/TPI dose reduction, baseline laboratory values, tumor markers, PFS, and OS.

BRAF and microsatellite instability (MSI) status were also reviewed when available. Missing data were summarized, and analyses were conducted using available cases (complete-case analysis) without performing imputation.

Adverse events and treatment exposure were reviewed descriptively using CTCAE version 5.0; however, these analyses were not predefined primary objectives.

### Clinical endpoints

The primary endpoint was PFS, and the secondary endpoint was OS. Treatment response was assessed by the attending physicians using computed tomography (CT) scans, clinical symptoms, and tumor marker trends.

### Definitions of PFS and OS

PFS was defined as the time from the first administration of FTD/TPI plus BEV to documented disease progression or death from any cause. OS was defined as the time from the first administration to death from any cause.

In this retrospective setting, imaging assessments (mainly contrast-enhanced CT) were generally performed every 3–6 months. Progression was primarily determined radiologically, with clinical deterioration or consistent tumor marker elevation considered supportive evidence when imaging was unavailable.

Patients without documented progression were censored at the date of the last disease assessment or follow-up.

### Statistical analysis

Continuous variables are expressed as medians with interquartile ranges (IQRs), and categorical variables are expressed as frequencies and percentages. Comparisons between the High-Alb and Low-Alb groups were made using the Mann–Whitney U test or Fisher’s exact test. Markedly elevated values of tumor markers (CEA and CA19-9) were retained in the analysis without truncation. The median PFS and OS were estimated using the Kaplan–Meier method, and survival curves were compared using the log-rank test. To evaluate the prognostic impact of baseline Alb, ROC curve analysis was used to determine the optimal cutoff value for Alb with reference to the median PFS. Additionally, sensitivity analyses were conducted to examine the robustness of results. First, patients were dichotomized based on the conventional clinical cutoff for hypoalbuminemia (3.5 g/dL). Second, Alb levels were stratified into quartiles based on the cohort distribution to assess potential dose-response trends in treatment efficacy.

To further identify independent predictors of PFS, Cox proportional hazards models were constructed. Based on prior studies, established prognostic factors for colorectal cancer (age, primary tumor site, metastatic sites, and LDH) [16, 21, 22] were considered candidate variables. Continuous variables such as Alb, LDH, and CRP were dichotomized based on ROC-derived cutoff values. Moreover, the NLR and the CRP-to-Alb ratio (CAR) were assessed in exploratory sensitivity analyses to further examine inflammation-related effects. Both indices were dichotomized at the cohort median to maintain analytical simplicity and to avoid model overfitting.

Differences in PFS among quartile groups were assessed using the log-rank test. A two-sided p-value of < 0.05 was considered statistically significant. Statistical analyses were performed using EZR (version 1.68; Saitama Medical Center, Jichi Medical University, Saitama, Japan).

### Ethical considerations

This study was conducted in compliance with the “Ethical Guidelines for Medical Research Involving Human Subjects” and the “Appropriate Handling of Personal Information by Medical and Nursing Care Providers” guidelines. Ethical approval was obtained from the Ethics Review Committee of Fukuyama Medical Center (approval number: ERBP2024005).

## Results

### ROC analysis of Alb levels for PFS

The median PFS of the study cohort was 3.7 months (IQR: 2.3–8.2 months). ROC curve analysis of baseline Alb levels for predicting PFS ≥ median identified an optimal cutoff of 3.7 g/dL, with an area under the curve (AUC) value of 0.740 (95% CI: 0.618–0.862), a sensitivity of 0.806, and a specificity of 0.697. Accordingly, patients were divided into the High-Alb (≥3.7 g/dL) and Low-Alb ( < 3.7 g/dL) groups.

### Baseline clinical characteristics of participants

A total of 69 patients were included (Fig. 1). The median age was 69 years (IQR: 60–74 years), and 35 patients (48.4%) were female. Notably, 67 patients (96.8%) had a performance status of 0 or 1 (Table 1). BRAF and MSI data were missing in 11.6% and 7.2% of patients, respectively. Given the low proportion of missing data and the exclusion of these variables from the multivariable analyses, complete-case analysis was performed. Right-sided tumors were observed in 26 patients (37.3%), and 51 patients (73.9%) had undergone primary resection. Prior therapies included BEV in 67 patients (97.1%), RAM in 30 (43.5%), and anti-epidermal growth factor receptor antibodies in 24 (34.8%). FTD/TPI plus BEV was administered as second- or third-line therapy in 38 patients (55.1%). Based on the Alb cutoff value, 39 patients were classified into the High-Alb group.

**Table 1:** Baseline characteristics of the study cohort

Characteristic	Overall (*N* = 69)		High-Alb group (*n* = 39)		Low-Alb group (*n* = 30)		p-value
Age, years	69	(60–74)	66	(57–73)	70	(67–74)	0.046
Sex, female; number (%)	35	(48.4%)	22	(56.4%)	13	(43.3%)	0.366
ECOG-PS (0–1); number (%)	67	(96.8%)	39	(100.0%)	28	(93.3%)	0.185
BMI (kg/m^2^)	22.7	(19.8–23.9)	22.7	(19.7–24.1)	22.9	(20.4–23.8)	0.904
KRAS mutation. number (%)	43	(59.7%)	26	(66.7%)	17	(56.7%)	0.457
BRAF status; number (%)							
Mutant	1	(1.4%)	0	(0.0%)	1	(3.3%)	—
Wild-type	60	(87.0%)	34	(87.2%)	26	(86.7%)	
Missing or Unknown	8	(11.6%)	5	(12.8%)	3	(10.0%)	
MSI status; number (%)							
High-MSI	1	(1.4%)	1	(2.6%)	0	(0.0%)	—
Stable or low MSI	63	(91.3%)	36	(92.3%)	27	(90.0%)	
Missing or Unknown	5	(7.2%)	2	(5.1%)	3	(10.0%)	
Liver metastasis; number (%)	47	(68.1%)	27	(69.2%)	20	(66.7%)	1.000
Lung metastasis; number (%)	38	(55.1%)	24	(61.5%)	14	(46.7%)	0.234
Peritoneal dissemination; number (%)	22	(31.9%)	13	(33.3%)	9	(30.0%)	0.800
Tumor location: Rectum (vs. colon); number (%)	17	(24.6%)	11	(28.2%)	6	(20.0%)	0.575
Tumor location: Right-sided (vs. left-sided); number (%)	26	(37.7%)	12	(30.8%)	14	(46.7%)	0.215
History of primary tumor resection; number (%)	51	(73.9%)	32	(82.1%)	19	(63.3%)	0.101
Prior bevacizumab treatment; number (%)	67	(97.1%)	37	(94.9%)	30	(100.0%)	0.501
Prior ramucirumab treatment; number (%)	30	(43.5%)	17	(43.6%)	13	(43.3%)	1.000
Prior anti-EGFR antibody treatment; number (%)	24	(34.8%)	12	(30.8%)	12	(40.0%)	0.455
Treatment line: 2nd or 3rd line (vs. ≥4th line); number (%)	38	(55.1%)	23	(59.0%)	15	(50.0%)	0.476
Initial dose reduction of FTD/TPI; number (%)	28	(40.6%)	12	(30.8%)	16	(53.3%)	0.084
Treatment schedule: Standard (days 1–5 & 8–12)	53	(76.8%)	29	(74.4%)	24	(80.0%)	0.775
Laboratory data							
White blood cell count,/µL	5,400	(4,200–7,500)	5,000	(4,200–6,300)	7,000	(4,100–8,800)	0.078
Lymphocyte count,/µL;	1,350	(970–1,625)	1,267	(1,005–1,600)	1,422	(912–1,762)	0.650
Hemoglobin, g/dL	11.8	(10.5–12.6)	12.0	(11.3–15.5)	11.1	(10.3–12.4)	0.017
Platelet, ×10^4^/µL	19.7	(13.8–24.3)	19.7	(15.5–23.5)	18.9	(12.5–27.8)	0.932
AST, U/L	29	(20–43)	24	(20–33)	34	(25–61)	0.003
ALT, U/L	21	(13–30)	19	(12–25)	25	(15–37)	0.045
Total bilirubin, mg/dL	0.6	(0.4–0.8)	0.6	(0.5–0.7)	0.6	(0.4–0.9)	0.937
LDH, U/L	255	(201–423)	237	(195–299)	372	(224–651)	0.016
Creatinine, mg/dL	0.74	(0.60–0.87)	0.7	2(0.60–0.82)	0.78	(0.68–0.88)	0.260
CRP, mg/dL	0.28	(0.12–1.27)	0.15	(0.10–0.79)	0.67	(0.20–2.26)	0.002
CEA, ng/mL	81.10	(21.15–427.04)	61.61	(9.24–269.85)	125.57	(32.21–724.86)	0.163
CA19-9, U/mL	251.97	(13.24–1,830.61)	210.30	(14.74–825.55)	382.31	(15.42–6,945.99)	0.268

Data are presented as medians (interquartile range [IQR], 25th–75th percentile) or as numbers (percentages). * *p* < 0.05 was considered statistically significant

Abbreviations: ECOG-PS, Eastern Cooperative Oncology Group performance status scale; BMI, body mass index; MSI, microsatellite instability; AST, aspartate aminotransaminase; ALT, alanine aminotransferase; LDH, lactate dehydrogenase; CRP, C-reactive protein; CEA, carcinoembryonic antigen; CA19-9, cancer antigen 19-9

### Comparison of clinical characteristics between the groups

Patients in the High-Alb group were significantly younger (66 [IQR: 57–73] years vs. 70 [IQR: 67–74] years, *p* = 0.046) and had higher hemoglobin levels (12.0 [IQR: 11.3–15.5] g/dL vs. 11.1 [IQR: 10.3–12.4] g/dL, *p* = 0.017) than those in the Low-Alb group. By contrast, LDH levels (237 [IQR: 195–299] U/L vs. 372 [IQR: 224–651] U/L, *p* = 0.016) and CRP levels (0.15 [IQR: 0.10–0.79] mg/dL vs. 0.67 [IQR: 0.20–2.26] mg/dL, *p* = 0.002) were significantly lower in the High-Alb group than in the Low-Alb group (all *p* < 0.05; Table 1).

### Association between Alb levels and survival

**Fig. 2:**
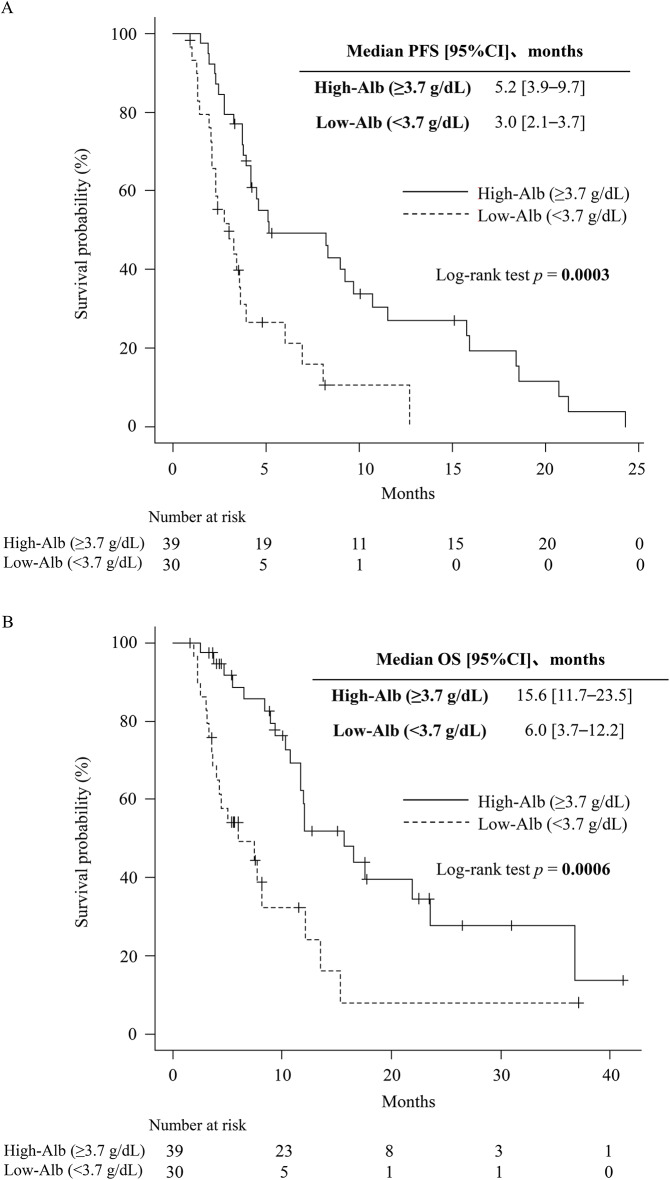
Kaplan–Meier curves (**A**) Kaplan–Meier curves for progression-free survival (PFS) stratified by baseline serum albumin (Alb) levels. Patients were categorized into High-Alb (≥3.7 g/dL) and Low-Alb (<3.7 g/dL) groups. The High-Alb group demonstrated significantly longer PFS than the Low-Alb group (median: 5.2 vs. 3.0 months; *p* < 0.001, log-rank test). (**B**) Kaplan–Meier curves for overall survival (OS) stratified by baseline serum Alb levels. The High-Alb group showed significantly longer OS than the Low-Alb group (median: 15.6 vs. 6.0 months; *p* < 0.001, log-rank test)

**Fig. 3:**
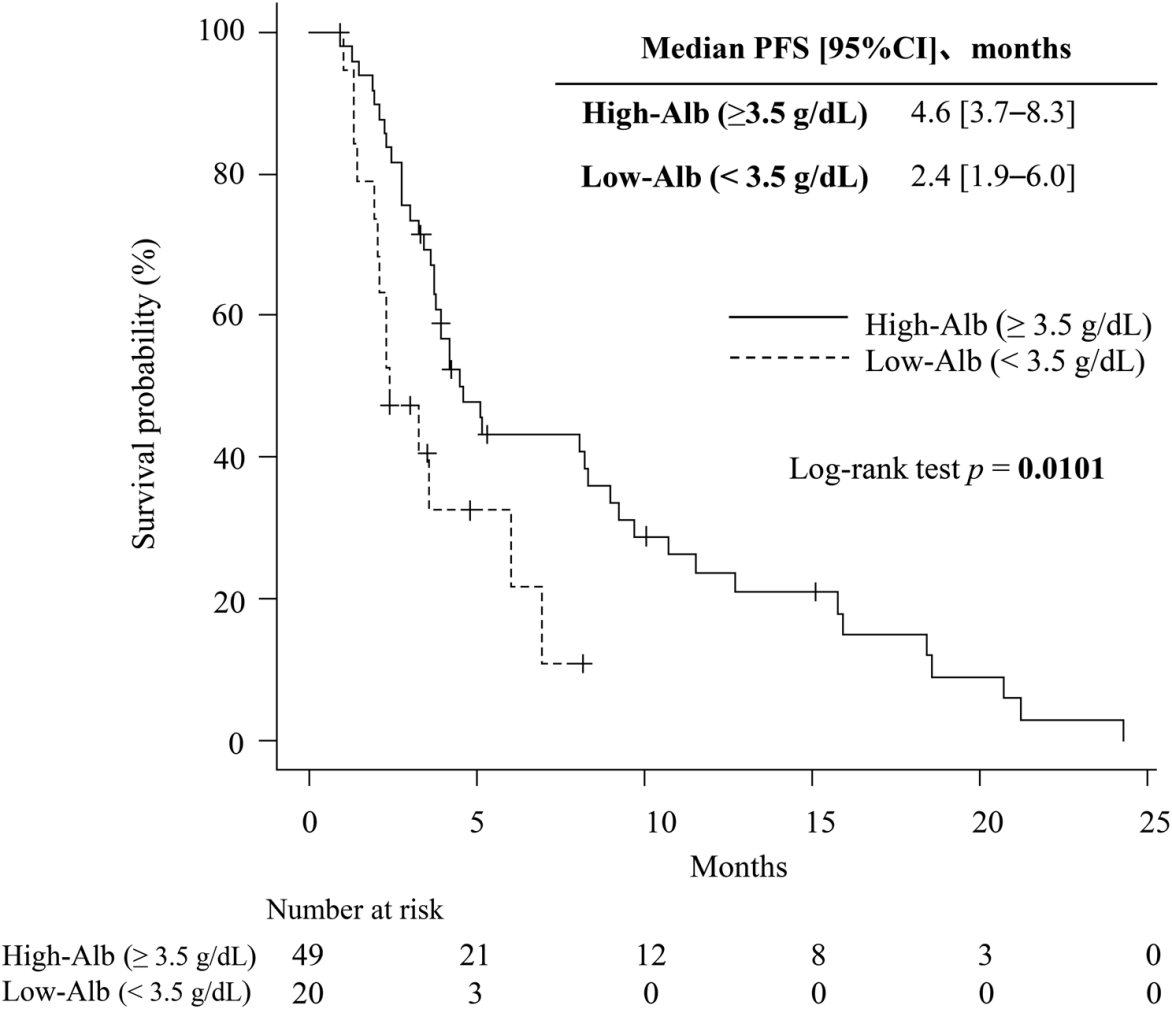
Kaplan–Meier curves for progression-free survival (PFS) stratified by baseline serum albumin using hypoalbuminemia cutoff. Patients were categorized into High-albumin (Alb) ( > 3.5 g/dL) and Low-Alb (≤3.5 g/dL) groups. The High-Alb group demonstrated significantly longer pfs than the Low-Alb group (median: 4.6 vs. 2.4 months; *p* = 0.007, log-rank test)

Patients in the High-Alb group had significantly longer PFS (median: 5.2 vs. 3.0 months; Fig. 2A) and OS (median: 15.6 vs. 6.0 months; Fig. 2B) than those in the Low-Alb group. In a sensitivity analysis using the conventional cutoff for hypoalbuminemia (3.5 g/dL), patients with Alb ≥ 3.5 g/dL had significantly longer PFS than those with Alb < 3.5 g/dL (median: 4.6 vs. 2.4 months; *p* = 0.010, log-rank test; Fig. 3).

When Alb levels were further divided into quartiles (Q1: ≤ 3.4 g/dL, Q2: 3.5–3.7 g/dL, Q3: 3.8–4.1 g/dL, and Q4: ≥ 4.2 g/dL), the median PFS gradually increased with higher Alb levels (2.4, 3.9, 4.6, and 5.2 months, respectively).

Although the difference among the four groups did not reach statistical significance (*p* = 0.051, log-rank test), a consistent trend toward longer PFS with increasing Alb levels was observed.

### Safety

Adverse events were evaluated in accordance with CTCAE criteria. Grade ≥3 neutropenia was observed in 22 of 39 patients (56.4%) in the high-Alb group and in 12 of 30 patients (40.0%) in the low-Alb group, with no significant difference observed between groups (*p* = 0.336). No treatment interruptions due to adverse events were observed in either group. (Three patients discontinued treatment owing to adverse events and were excluded from the analysis in accordance with the study criteria.) Therefore, detailed safety analysis was not conducted, as we primarily focused on efficacy outcomes.

### ROC analysis of LDH and CRP levels for predicting treatment efficacy

ROC analysis of serum LDH and CRP levels for predicting PFS ≥ median identified optimal cutoff values of 338 U/L and 1.3 mg/dL, respectively. For LDH, the AUC was 0.605 (95% CI: 0.467–0.742), with a sensitivity of 80.6% and specificity of 45.5%. For CRP, the AUC was 0.652 (95% CI: 0.522–0.782), with a sensitivity of 88.9% and specificity of 36.4%.

### Exploratory analysis of predictors of PFS

To identify the predictors of PFS, two Cox proportional hazards models were constructed (Table 2). In Model 1—adjusted for age and primary tumor site [20, 23]—Alb ≥3.7 g/dL was the only significant factor and was associated with prolonged PFS (HR: 0.35, 95% CI: 0.19–0.63, *p* < 0.001). In Model 2—adjusted for peritoneal dissemination and LDH ≥338 U/L [21, 22]—both Alb ≥3.7 g/dL (HR: 0.40, 95% CI: 0.22–0.73, *p* = 0.003) and LDH ≥338 U/L (HR: 2.31, 95% CI: 1.28–4.32, *p* = 0.009) were independently associated with PFS. To further explore the potential impact of systemic inflammation, additional multivariate models incorporating inflammation-based indices were constructed (Model 3a and Model 3b; Table 2). Neither the NLR nor the CAR showed a significant association with PFS, whereas higher Alb (≥3.7 g/dL) remained independently associated with longer PFS. These findings suggest that the prognostic effect of Alb was not solely attributable to inflammatory status.

**Table 2:** Multivariate and univariate Cox analyses of baseline factors associated with progression-free survival

	Univariate model			Multivariate model 1 (Minimal)			Multivariate model 2 (Clinically enriched)			Multivariate model 3a (Fully adjusted, inflammation-adjusted)			Multivariate model 3b (Fully adjusted, CAR-based)		
	HR	(95% CI)	p-value	HR	(95% CI)	p-value	HR	(95% CI)	p-value	HR	(95% CI)	p-value	HR	(95% CI)	p-value
LDH, ≥338 U/L	2.68	1.47–4.88	0.001	2.31	1.24–4.32	0.009	－	－	－	2.24	0.19–4.23	0.013	2.62	1.28–5.41	0.009
Peritoneal dissemination, yes	1.11	0.62–2.00	0.729	1.40	0.76–2.56	0.281	－	－	－	－	－	－	－	－	－
Primary tumor location: Right-sided, yes	1.58	0.88–2.84	0.129	－	－	－	－	－	－	－	－	－	－	－	－
Age (≥65 years), yes	1.22	0.70–2.12	0.489	－	－	－	1.44	0.81–2.55	0.216	－	－	－	－	－	－
Treatment line: 2nd or 3rd (vs. ≥4th), yes	0.75	0.44–1.27	0.283	－	－	－	0.82	0.48–1.43	0.494	－	－	－	0.91	0.52–1.57	0.726
CRP, ≥1.3 mg/dL	2.10	1.12–3.93	0.020	－	－	－	－	－	－	－	－	－	－	－	－
NLR, ≥2.58 (median)	1.15	0.68–1.95	0.601	－	－	－	－	－	－	0.95	0.55–1.63	0.858	－	－	－
CAR × 10^3^, ≥76.5 (median)	1.48	0.87–2.52	0.151	－	－	－	－	－	－	－	－	－	0.98	0.52–1.83	0.948
Albumin, ≥3.7 g/dL	0.36	0.20–0.64	< 0.001	0.396	0.22–0.73	0.003	0.35	0.19–0.62	< 0.001	0.42	0.23–0.76	0.004	－	－	－

*p* < 0.05 was considered statistically significant

Abbreviations: LDH, lactate dehydrogenase; CRP, C-reactive protein; NLR, neutrophil-to-lymphocyte ratio; CAR, C-reactive protein-to-albumin ratio; HR, hazard ratio; CI, confidence interval

## Discussion

In this study, we found that baseline serum Alb levels at the initiation of FTD/TPI plus BEV therapy were significantly associated with PFS and OS. Furthermore, patients with pretreatment Alb levels of ≥3.7 g/dL tended to experience more favorable outcomes with FTD/TPI plus BEV therapy. These findings suggest that Alb may serve as a simple and potentially useful marker for predicting the treatment response of FTD/TPI plus BEV in clinical practice.

The median PFS in our study cohort was 3.7 months, which was somewhat shorter than the 5.6 months reported in a previous trial [5]. In that study, the median age of patients was 62 years, and 92% received third-line therapy; however, the median age of patients in the present study was 69 years, and 45% received fourth-line or later therapy. These differences suggest that our study population was older and more heavily pretreated, potentially reflecting a clinical setting closer to real-world practice. Within this cohort, the median PFS in the High-Alb group was 5.2 months, approaching the outcome reported in a previous study [5]. By contrast, patients in the Low-Alb group experienced more limited PFS benefit.

Several factors may account for these findings. First, reduced Alb levels often reflect cancer progression and systemic inflammation, which may be associated with diminished treatment responsiveness. Previous studies have shown that the CAR ratio is correlated with poor PFS and OS in patients with CRC [24], and serum Alb has been reported as an independent prognostic factor for CRC [25–27]. Cancer-associated inflammation reduces hepatic Alb synthesis via cytokines such as interleukin-6, and persistent hypoalbuminemia may contribute to cancer progression and mortality [28–30]. Although Alb levels can be influenced by factors other than cancer [31], our findings indicate that baseline Alb levels can provide clinically useful insights into treatment response to FTD/TPI plus BEV. In Cox proportional hazards models, Alb levels ≥3.7 g/dL were significantly associated with prolonged PFS, and the association remained significant in multiple models. Even after adjustment for inflammation-based indices such as NLR and CAR, Alb remained independently associated with favorable PFS, suggesting its robustness as a prognostic marker. Although the reference range for adult serum Alb typically ranges from 3.5 to 5.0 g/dL—with levels below 3.5 g/dL generally considered hypoalbuminemia [32, 33]—institutional ranges vary. At our institution, the normal range for serum Alb is 4.1–5.1 g/dL; therefore, the cutoff value of 3.7 g/dL identified in this study was deemed clinically reasonable. Moreover, Alb may act as an integrative marker reflecting the nutritional status, inflammatory activity, treatment responsiveness, and overall condition of the patient. This interpretation is supported by the significantly lower CRP and LDH levels observed in the High-Alb group. As CRP reflects systemic inflammation and LDH reflects tumor burden and metabolic activity [13–15], lower values of these markers suggest that patients in the High-Alb group may have relatively attenuated inflammation and tumor progression. Accordingly, higher Alb levels may indicate a better nutritional status and reflect a background of attenuated inflammatory activity and tumor burden, which could contribute to more favorable treatment outcomes.

A second possible explanation is that hypoalbuminemia or elevated LDH levels may have affected the pharmacokinetics and pharmacodynamics of BEV. In this study, PFS in the High-Alb group (5.2 months) was comparable to that reported for the BEV combination group in the SUNLIGHT trial (5.6 months) [5], despite differing patient backgrounds. Conversely, the Low-Alb group achieved a PFS of 3.0 months, closer to that of the FTD/TPI monotherapy group (2.4 months) in the same trial. This raises the possibility that low Alb levels may alter BEV pharmacokinetics, thereby attenuating its efficacy. Prior studies have suggested that even mild proteinuria—defined as a urinary protein-to-creatinine ratio of ≤ 1 g/dL—may lower BEV concentrations and reduce its therapeutic effect [34]. Although urinary protein levels were not assessed in the present study, patients in the Low-Alb group may have had chronic, low-grade protein loss, potentially contributing to decreased Alb levels and reduced BEV exposure.

In addition, the expression of vascular endothelial growth factor is influenced by hypoxia and inflammation, which may affect the efficacy of BEV [35]. Notably, prior real-world analyses suggest worse outcomes with hypoalbuminemia under TAS-102 therapy [36]; Moreover, hypoalbuminemia may also affect the pharmacology of the FTD/TPI regimen itself. Trifluridine is highly protein-bound ( > 96% Alb binding), whereas tipiracil exhibits minimal protein binding ( < 8%). Under hypoalbuminemic conditions—frequently associated with inflammation or malnutrition—the free fraction of trifluridine may increase, accelerating its distribution or elimination, thereby reducing effective exposure at the tumor site [37]. In CRC, breast cancer, and renal cell carcinoma, BEV has been reported to prolong PFS and OS, particularly in patients with low levels of systemic inflammation [38–40]. Although evidence is more limited for non–small cell lung cancer, some reports have shown associations between BEV efficacy and inflammatory markers [41, 42]. Hoang et al. reported that hypoalbuminemia was associated with worse PFS and OS [41]. In the present study, the Low-Alb group also exhibited lower hemoglobin and higher CRP and LDH levels, consistent with persistent inflammation, suggesting reduced responsiveness to BEV. Furthermore, LDH was identified as a predictive factor in multivariate analysis. A meta-analysis of LDH and BEV in CRC confirmed significant associations with both PFS and OS [43], and other studies have proposed LDH as a potential predictive biomarker for anti-angiogenic therapy [44, 45]. Moreover, in the HORIZON I trial, Bar et al. reported that elevated LDH levels were significantly associated with shorter PFS [46]. Our results are broadly consistent with these prior findings.

Taken together, these observations suggest that serum Alb may be an important factor influencing the treatment outcomes of FTD/TPI plus BEV therapy through mechanisms involving systemic inflammation, tumor activity, and pharmacokinetics.

This study had some limitations. First, it was a single-center, retrospective study with a limited sample size, which may have led to insufficient statistical power to detect modest differences; therefore, the results should be interpreted with caution. Large-scale, prospective studies are needed to validate these findings. Second, response rates were not evaluated, preventing the direct assessment of treatment efficacy. Third, other prognostic factors—such as histological subtype and molecular alterations (e.g., BRAF V600E)—were not fully investigated; therefore, the possibility remains that the Low-Alb group included patients with a more aggressive disease. In addition, multiple between-group and exploratory comparisons were performed without multiplicity adjustment; therefore, these findings should be interpreted cautiously as exploratory and hypothesis-generating. Future prospective studies incorporating factors such as proteinuria, circulating drug exposure, and nutritional interventions are warranted to validate these findings.

## Conclusions

The findings of this study suggest that serum Alb may serve as a potential predictive factor for the efficacy of FTD/TPI plus BEV therapy in patients with mCRC. Initiating FTD/TPI plus BEV therapy before substantial deterioration of nutritional or inflammatory status associated with disease progression may help achieve more favorable clinical outcomes.

## Data Availability

The datasets used and/or analyzed during the current study are available from the corresponding author upon reasonable request.

## Abbreviations

Alb: Albumin
AUC: Area under the curve
BEV: Bevacizumab
BMI: Body mass index
CI: Confidence interval
CRC: Colorectal cancer
CRP: C-reactive protein
FTD/TPI: Trifluridine/tipiracil
HR: Hazard ratio
IQR: Interquartile range
LDH: Lactate dehydrogenase
mCRC: Metastatic colorectal cancer
OS: Overall survival
PFS: Progression-free survival
ROC: Receiver operating characteristic.
